# Relaxin and β-estradiol modulate targeted matrix degradation in specific synovial joint fibrocartilages: progesterone prevents matrix loss

**DOI:** 10.1186/ar1978

**Published:** 2006-06-19

**Authors:** Gihan Hashem, Qin Zhang, Takayuki Hayami, Jean Chen, Wei Wang, Sunil Kapila

**Affiliations:** 1 Department of Orthodontics and Pediatric Dentistry, University of Michigan, 1011 North University Avenue, Ann Arbor, MI 48109-1078, USA

## Abstract

Relaxin, a 6-kDa polypeptide hormone, is a potent mediator of matrix turnover and contributes to the loss of collagen and glycosaminoglycans (GAGs) from reproductive tissues, including the fibrocartilaginous pubic symphysis of several species. This effect is often potentiated by β-estradiol. We postulated that relaxin and β-estradiol might similarly contribute to the enhanced degradation of matrices in fibrocartilaginous tissues from synovial joints, which may help explain the preponderance of diseases of specific fibrocartilaginous joints in women of reproductive age. The objective of this study was to compare the *in vivo *effects of relaxin, β-estradiol, and progesterone alone or in various combinations on GAG and collagen content of the rabbit temporomandibular joint (TMJ) disc fibrocartilage, knee meniscus fibrocartilage, knee articular cartilage, and the pubic symphysis. Sham-operated or ovariectomized female rabbits were administered β-estradiol (20 ng/kg body weight), progesterone (5 mg/kg), or saline intramuscularly. This was repeated 2 days later and followed by subcutaneous implantation of osmotic pumps containing relaxin (23.3 μg/kg) or saline. Tissues were retrieved 4 days later and analyzed for GAG and collagen. Serum relaxin levels were assayed using enzyme-linked immunosorbent assay. Relaxin administration resulted in a 30-fold significant (*p *< 0.0001) increase in median levels (range, approximately 38 to 58 pg/ml) of systemic relaxin. β-estradiol, relaxin, or β-estradiol + relaxin caused a significant loss of GAGs and collagen from the pubic symphysis and TMJ disc and of collagen from articular cartilage but not from the knee meniscus. Progesterone prevented relaxin- or β-estradiol-mediated loss of these molecules. The loss of GAGs and collagen caused by β-estradiol, relaxin, or β-estradiol + relaxin varied between tissues and was most prominent in pubic symphysis and TMJ disc fibrocartilages. The findings suggest that this targeted modulation of matrix loss by hormones may contribute selectively to degeneration of specific synovial joints.

## Introduction

The development and maintenance of cartilage entails active secretion of macromolecular glycosaminoglycans (GAGs) and collagens by chondrocytes, resulting in an organized extracellular matrix (ECM), which confers specific mechanical and physiologic properties to cartilage [1]. Chondrocytes also play a critical role in the normal remodeling of cartilaginous tissues by expressing tissue-degrading proteinases, primarily those belonging to the matrix metalloproteinase (MMP) family of enzymes [2]. This normal tissue turnover is regulated by many local and systemic agents, including peptide and steroid hormones, and entails the maintenance of a finely tuned balance between matrix synthesis and degradation. In degenerative joint diseases, an imbalance between synthesis and degradation of the ECM which results primarily from altered chondrocyte function leads to the net loss of tissue macromolecules and compromises joint function. Because several degenerative joint diseases have a high female-to-male preponderance, a regulatory role of specific sex hormones has been implicated in controlling the metabolism of these tissues [3-5]. This is particularly true of the highly prevalent diseases of the fibrocartilaginous temporomandibular joint (TMJ), which also have a high female-to-male predilection but which, unlike similar diseases of other joints which largely occur postmenopausally, are observed primarily in women of reproductive age [6,7]. These observations have led to the hypothesis that female hormones, including estrogen and relaxin, play a crucial role in predisposing women to TMJ diseases.

Relaxin H2, a 6-kDa polypeptide hormone that is structurally related to the insulin family of hormones which is primarily synthesized by the corpus luteum and placenta, is a known mediator of ECM remodeling in several reproductive tissues, including the uterus, cervix, ovary, breast, and the pubic symphysis [8-13]. Within the fibrocartilaginous pubic symphysis and cervix, relaxin plays an important role during parturition by mediating the remodeling necessary for the successful delivery and survival of pups [14,15]. Although the precise mechanisms for relaxin's modulation of matrix turnover have not been fully elucidated, it appears to exert these effects by mediating the synthesis and/or degradation of matrix macromolecules [12,16-18]. The latter mechanism likely involves relaxin's induction of several members of MMPs [5,19-21].

In several species, including guinea pigs, mice, bats, and humans, relaxin induces the transformation of pubic joint fibrocartilage into a flexible and elastic interpubic ligament during pregnancy [17]. These changes in the characteristics of the pubic symphysis result from a decrease in collagen content caused by relaxin, which in some species is potentiated by the prior or concurrent administration of estrogen [12,13,18]. Because of the matrix remodeling effects of relaxin and β-estradiol on the fibrocartilaginous pubic symphysis, it is plausible that other cartilages within synovial joints may be among the other presumptive, yet-to-be-proven, non-reproductive target sites of the tissue-remodeling activity of these hormones. Indirect evidence for such a modulation of matrix turnover of cartilage by relaxin is provided by studies showing that relaxin produces a dose-dependent induction of MMPs collagenase-1 (MMP-1) and stromelysin-1 (MMP-3) in fibrocartilaginous cells of the TMJ disc [5]. Priming of these cells with β-estradiol potentiated their MMP-inductive response to relaxin, resulting in the maximal expression of collagenase-1 and stromelysin-1 at relaxin concentrations that were 10- to 100-fold lower in β-estradiol-primed cells than in unprimed cells. These observations on isolated fibrocartilaginous cells are consistent with observations that treatment with estrogen in some, but not all, species or reproductive tissues further enhances the relaxin-mediated induction of MMPs [9,10,22] and the loss of ECM [12,16,20,23]. Pertinent to synovial joints, *in vitro *studies [21] on TMJ fibrocartilaginous explants have shown that the induction of collagenase-1 and stromelysin-1 by relaxin is accompanied by a loss of collagen and GAGs.

Previous findings on TMJ fibrochondrocytes [5] and fibrocartilaginous explants [21] have raised the possibility that reproductive hormones, including relaxin, interplay with estrogen and progesterone on the remodeling of joint ECM. These data [5,21] also lend support to the contention that specific sex hormones play an important role in the physiologic or pathologic remodeling of cartilaginous tissues in select synovial joints such as the TMJ by modulating the turnover of fibrocartilaginous ECM via induction of MMPs. Although this postulate is supported by *in vitro *studies [5,21], no *in vivo *data are currently available to validate this hypothesis. Moreover, whether these hormones specifically target matrix turnover in the TMJ fibrocartilage more profoundly than that in similar tissues of other joints has not been demonstrated. Therefore, the aim of this study was to determine the *in vivo *effects of relaxin, β-estradiol, and progesterone either alone or in various combinations on the total GAG and collagen content in the rabbit TMJ disc fibrocartilage and to compare these responses with those of knee meniscus fibrocartilage and articular cartilage. Because the effects of these hormones on pubic symphyseal collagen have been characterized previously [12], we used this tissue as a positive control.

## Materials and methods

### Experimental design and animal procedures

All animal experiments were conducted with the approval of the Institutional Review Board of the University of California San Francisco. Bilaterally ovariectomized or sham-operated 18-week-old female New Zealand white rabbits were obtained from Covance Frams (Covance Inc., Princeton, NJ, USA) and housed in a controlled environment with free access to food and water. Two weeks later, considered day 0 of the experiments, all rabbits in the experimental groups received an intramuscular injection of 1 ml saline solution with 20 ng/kg body weight of β-estradiol (Sigma-Aldrich, St. Louis, MO, USA) and/or 5 mg/kg body weight of progesterone (Sigma-Aldrich) while rabbits in normal control and sham-operated groups were administered 1 ml normal saline intramuscularly. The specific concentrations of the two hormones were selected because these doses have been shown to result in systemic levels similar to those found physiologically in cycling women [24,25].

Two days later, the rabbits were anesthetized with 40 mg/kg of ketamine hydrochloride (Parke-Davis Inc., Morris Plains, NJ, USA) and 3 to 5 mg/kg of xylazine (Ruby Lab, Rockville Centre, NY, USA) for administration of β-estradiol and/or progesterone or normal saline as described for day 0 implantation of osmotic pumps and blood collection. Osmotic pumps (2ML1; Durect Corporation, Cupertino, CA, USA) were loaded with 2 ml of normal saline or 2 ml of 23.3 μg/kg body weight of recombinant human relaxin (Connetics Corporation, Palo Alto, CA, USA). This concentration of relaxin has been shown to result in serum levels similar to those found in women [26]. The pumps were implanted via a dermal incision of approximately half an inch made in the upper back of the animals, and approximately 2.5 inches of skin was freed with a blunt forceps from the underlying tissues; the pump was inserted into this cavity, and the incision was sutured. Approximately 5 ml of blood was collected, and the serum was processed and stored for further analysis.

The procedures and administration of hormones resulted in eight groups of rabbits, each with six to eight rabbits, as follows: (a) sham-operated controls receiving saline, (b) ovariectomized normal controls receiving saline, (c) ovariectomized rabbits receiving β-estradiol, (d) ovariectomized rabbits receiving progesterone, (e) ovariectomized rabbits receiving relaxin, (f) ovariectomized rabbits receiving β-estradiol and relaxin, (g) ovariectomized rabbits receiving progesterone and relaxin, and (h) ovariectomized rabbits receiving β-estradiol, progesterone, and relaxin.

### Tissue retrieval and serum collection

At days 2 and 6, the rabbits were anesthetized as described above, blood was collected from an ear vein into a coagulation tube, and the serum was retrieved through centrifugation at 1,100 *g *for 10 minutes. The serum aliquots were stored at -70°C until further analysis.

On day 6, while under anesthesia, the rabbits were sacrificed using 1 ml of 50 mg/ml of Nembutal (Abbott Laboratories, Abbott Park, IL, USA) injected into the pericardium, and the pubic symphysis and the bilateral TMJ discs, knee menisci, and articular cartilage were retrieved under sterile conditions. The tissues were cleaned of any fat, muscle, or bone, patted dry, lyophilized in a SpeedVac (Labconco Corporation, Kansas City, MO, USA), and weighed (dry weight). Tissues were digested in 3 mg/ml pepsin (Sigma-Aldrich) in 0.5 M acetic acid at 45°C until completely digested and were used for determination of GAG and collagen content.

### Quantification of GAG concentrations

Total GAG in tissue was evaluated using the dimethylmethylene blue (DMMB) dye assay. Briefly, 50 μl of the pepsin digest was mixed with 200 μl DMMB dye (Sigma-Aldrich) in each well of a 96-well plate. The optical density (OD) was determined using a plate reader (SPECTRAmax PLUS; Molecular Devices, Cupertino, CA, USA) at a wavelength of 535 nm. The GAG content (μg/ml) was determined by comparing the OD of the sample against a standard curve prepared from 0 to 2.5 μg/well of bovine chondroitin sulfate A (Sigma-Aldrich). The tissue GAG concentrations were calculated by normalizing the GAG content to the dry weight of the tissues.

### Quantification of total collagen concentrations

Pepsin digest (200 μl) was mixed with 1 ml of Sircol dye reagent (Biocolor Ltd., Newtownabbey, UK), incubated at room temperature for 30 minutes, and centrifuged at 10,000 *g *to separate the unbound dye from the collagen-bound dye. After removal of the unbound dye, 1 ml of the alkaline reagent was added to the collagen-dye complex and vortexed. Aliquots (200 μl) were transferred to the 96-well plates, and the absorbance was determined at 550 nm with a microtiter plate reader. The collagen concentration (μg/ml) was determined against a collagen standard curve, and the tissue collagen content was standardized to the total disc dry weight.

### Determination of serum relaxin levels

Systemic relaxin concentration in serum was measured using a commercially available enzyme-linked immunosorbent assay kit (American Laboratory Products Company, Windham, NH, USA) according to the manufacturer's instructions. Briefly, 100 μl of samples or standards (0 to 250 pg/ml) was pipetted in duplicate into wells coated with antibody to relaxin and was incubated at 4°C for 12 hours. After further washes, the biotinylated anti-relaxin antibody was added and incubated for 2 hours at 4°C. After the wells were washed again, horseradish peroxidase-streptavidin was added, followed by incubation at 4°C for 1 hour. After washing, the color was developed using tetramethylbenzidine, the reaction was stopped with 4.5 N sulfuric acid, and the OD value was determined at 450 nm on a plate reader.

### Statistical analysis

GAG and collagen concentrations of the TMJ disc, knee meniscus, and articular cartilage in each rabbit were calculated as a mean value from bilateral samples. Data from six to eight rabbits in each group were expressed as mean ± standard deviation. Statistical analysis was performed using single-factorial analysis of variance, and inter-group differences were determined using Fisher's multiple comparison tests, with *p *< 0.05 being considered statistically significant. Systemic relaxin concentration of relaxin in each group was expressed as median with inter-quartile value and was analyzed by the non-parametric Kruskal-Wallis test, with a *p *< 0.05 being considered statistically significant.

## Results

### Relaxin administration increases systemic relaxin concentration, which is accentuated by β-estradiol priming and attenuated by progesterone treatment

**Figure 1:**
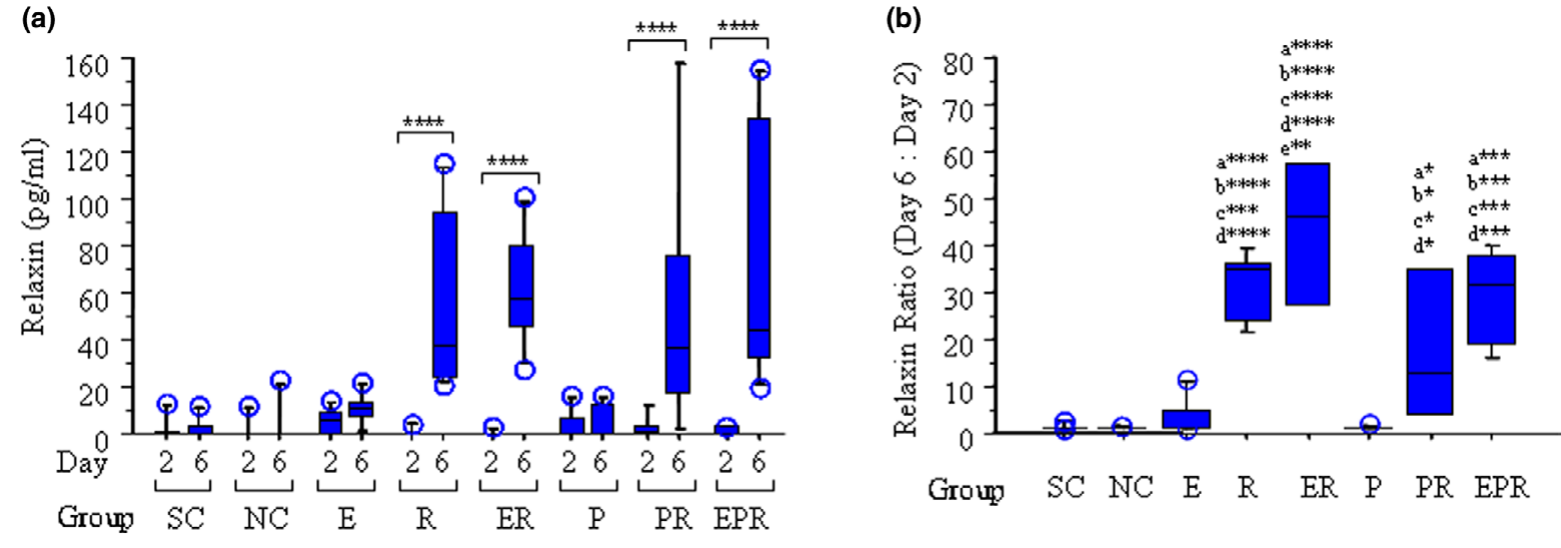
Relaxin (R) administration increases serum R concentrations, which is enhanced by β-estradiol (E) and attenuated by progesterone (P). Systemic R concentrations were assayed in serum from sham-operated controls (SC), ovariectomized normal controls (NC), and rabbits treated with E, R, E + R (ER), P, P + R (PR), and E + P + R (EPR) groups on day 2 and day 6, respectively, using an enzyme-linked immunosorbent assay as described. **(a)** Histograms of the median with inter-quartile value showed that there was no statistically significant difference in the basal levels of R on day 2 between any of the groups. Additionally, the SC, NC, E, and P groups showed no statistically significant differences in R concentrations between day 2 and day 6. R administration resulted in a significant increase of systemic R concentration on day 6 in R, ER, PR, and EPR groups compared with the corresponding basal levels of R on day 2. **(b)** The fold change in R was determined by normalizing the day-6 concentration to the corresponding day-2 concentration of R for each animal, and the median with inter-quartile value was plotted for each group of animals. The fold increase in R was significantly greater in R (35-fold), ER (46-fold), PR (13-fold), and EPR (32-fold) groups compared with that in SC, NC, E, and P groups. No statistically significant difference of R ratio was found between any of the R-treated groups, with the exception of ER versus PR groups (*p *< 0.01). Data were collected from a minimum of six rabbits in each group and expressed as median with inter-quartile value. (* < 0.05; ** < 0.01; *** < 0.001; **** < 0.0001; a, versus SC; b, versus NC; c, versus E; d, versus P; e, versus PR.)

On day 2, low to undetectable levels of endogenous relaxin were noted in all groups of rabbits (Figure 1a). Similarly, control rabbits implanted with osmotic pumps containing saline demonstrated low to non-detectable levels of relaxin on day 6. In contrast, all four groups of rabbits implanted with osmotic pumps containing relaxin showed substantial increases in systemic relaxin concentration on day 6. The median concentration was 38 pg/ml for rabbits that received relaxin alone, 58 pg/ml for those administered β-estradiol and relaxin, 37 pg/ml for rabbits receiving progesterone + relaxin, and 44 pg/ml for those administered progesterone + β-estradiol + relaxin. All these groups showed significantly greater systemic relaxin levels on day 6 than the corresponding basal relaxin levels on day 2.

The changes in systemic relaxin were further assessed by normalizing relaxin concentrations on day 6 to the corresponding basal level of relaxin on day 2 within each group (Figure 1b). Overall, there was a 30-fold increase in median levels of systemic relaxin in all four groups receiving relaxin, which is significantly higher than the changes in sham-operated and ovariectomized normal controls, and in rabbits receiving either β-estradiol or progesterone alone. The median fold increase in systemic relaxin concentration ranged from a low of 13-fold in the progesterone + relaxin group, followed by 32-fold in the β-estradiol + progesterone + relaxin group, 35-fold in the relaxin group, to a high of 46-fold in β-estradiol + relaxin group. The fold increase in systemic relaxin levels between all but one pair of the four groups receiving relaxin was not statistically significant. Interestingly, administration of relaxin to β-estradiol-primed rabbits produced a significantly greater (*p *< 0.01) fold increase in systemic relaxin concentrations as compared with the fold increase observed when relaxin was administrated to progesterone-primed rabbits.

### β-estradiol, relaxin, and β-estradiol + relaxin, but not progesterone, selectively causes the loss of GAGs from specific fibrocartilages

**Figure 2:**
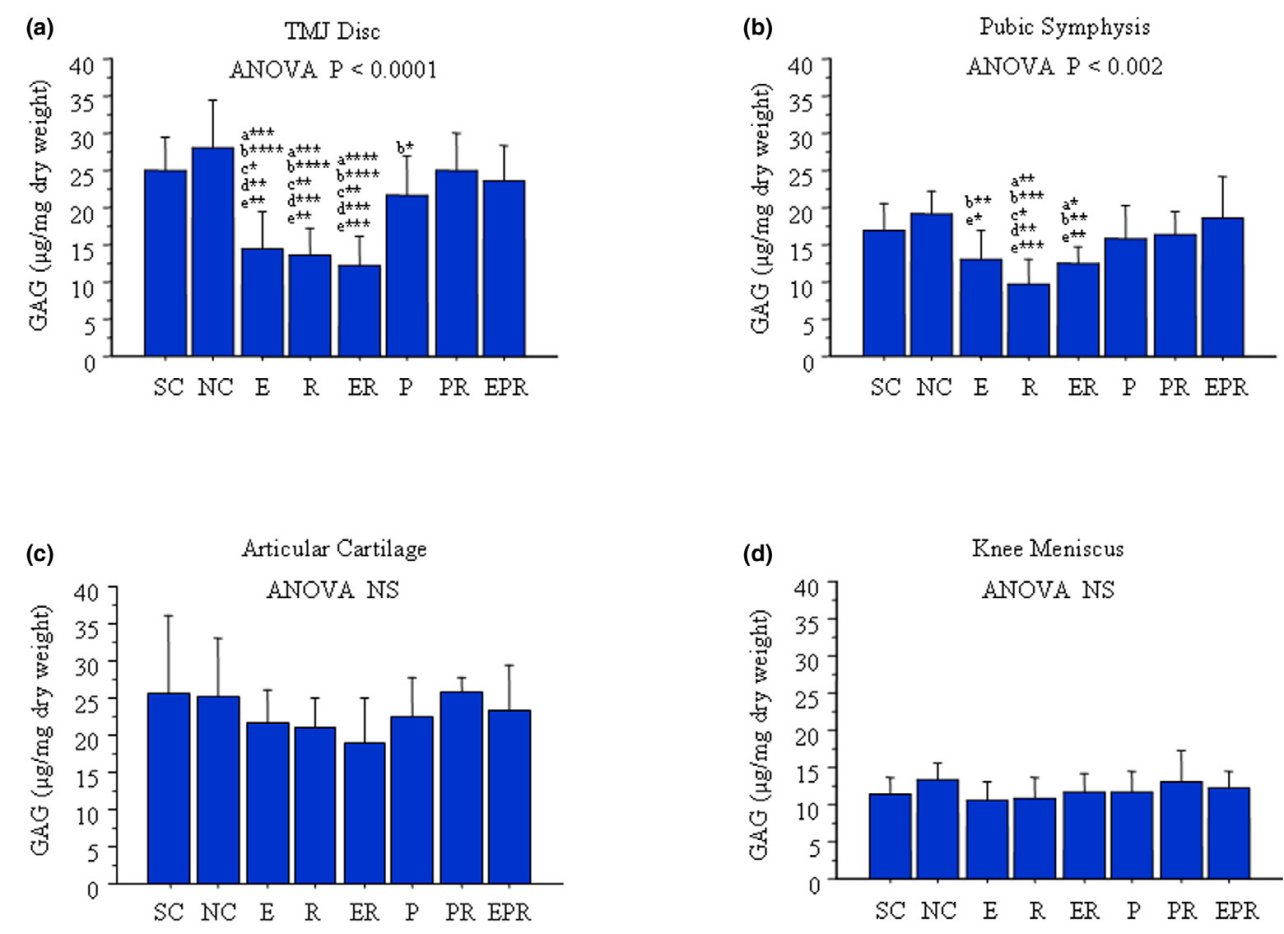
β-estradiol (E) or relaxin (R) treatment causes loss of GAGs from specific fibrocartilages while progesterone (P) inhibits this effect. Temporomandibular joint (TMJ) discs, pubic symphysis, knee meniscus, and articular cartilage were retrieved from control and hormone-treated rabbits, and total GAG content was determined by dimethylmethylene blue assay and normalized to the total dry weight for each sample. Histograms of the mean (± standard deviation) GAG concentration in sham-operated controls (SC), ovariectomized normal controls (NC), and rabbits treated with E, R, ER, P, P + R (PR), and E + P + R (EPR) groups were plotted. Ovariectomy had minimal effect on total GAGs present in any of these four tissues. E, R, and ER produced a significant reduction of GAGs in TMJ disc **(a)** and pubic symphysis **(b)** relative to SC and NC groups. P alone contributed to the maintenance of GAGs in all these tissues or prevented E-, R-, or ER-mediated loss of GAGs from both TMJ disc and pubic symphysis. None of these hormone treatments caused a significant change of GAGs in knee articular cartilage **(c)** and meniscus **(d)**. Data were collected from a minimum of six rabbits in each group. (* < 0.05; ** < 0.01; *** < 0.001; **** < 0.0001; a, versus SC; b, versus NC; c, versus P; d, versus PR; e, versus EPR.) ANOVA, analysis of variance; NS, not significant.

The sham-operated rabbits demonstrated varied basal concentrations of GAGs in each tissue type; these concentrations were highest in the TMJ disc and knee cartilage, followed by the pubic symphysis, and then the knee meniscus (Figure 2a–d). Ovariectomy had minimal effects on the total GAGs present in any of the four tissues. Exposure of the rabbits to various hormone treatments resulted in responses that varied among tissue types. β-estradiol or relaxin alone or β-estradiol + relaxin produced a statistically significant reduction of approximately 50% in TMJ disc GAG concentrations relative to sham-operated and ovariectomized control rabbits (Figure 2a). These hormones also contributed to a statistically significant reduction of GAGs in pubic symphyseal fibrocartilage, which at a 30% decrease was less marked than for the TMJ disc (Figure 2b). None of the hormone treatments caused any significant changes in GAG content of the knee articular cartilage (Figure 2c) or knee meniscus (Figure 2d).

Treatment with progesterone alone contributed to the maintenance of GAG concentrations in the TMJ disc and pubic symphysis fibrocartilages at levels found in sham-operated and ovariectomized control rabbits. Furthermore, administration of progesterone prevented the relaxin-mediated or β-estradiol + relaxin-mediated loss of GAGs from these tissues. Also, the combined administration of all three hormones maintained levels of GAG similar to that in sham-operated rabbits, suggesting that these hormones together produced a near physiologic effect on the joint tissues.

Together, these findings demonstrate varied responses of different fibrocartilaginous and hyaline cartilaginous tissues to relaxin, β-estradiol, and progesterone. We also show that progesterone has an important effect in minimizing the degradative effects of β-estradiol and relaxin on TMJ disc and pubic symphyseal GAG content.

### β-estradiol, relaxin, and progesterone have differential effects on the loss of collagen in fibrocartilaginous tissues

**Figure 3:**
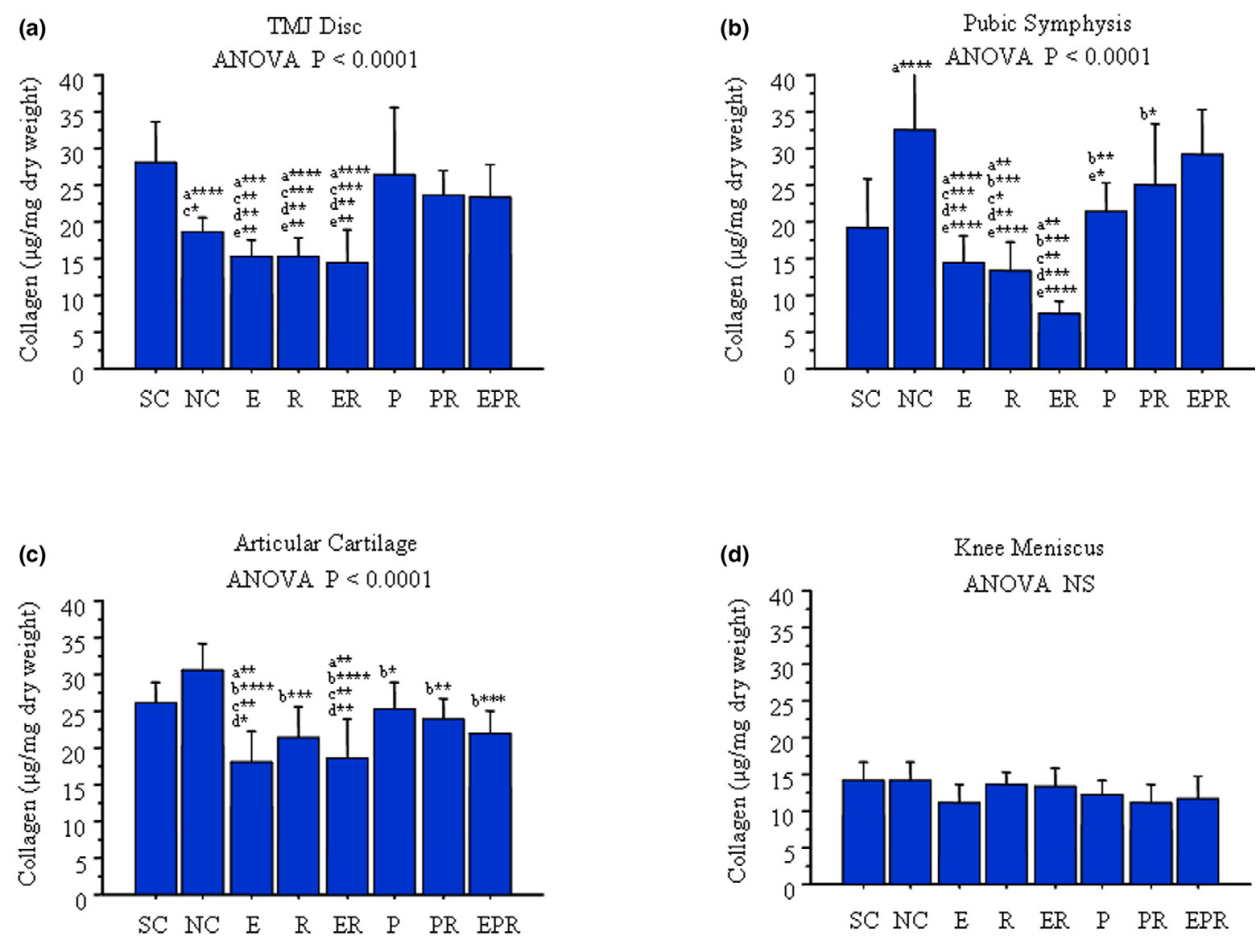
β-estradiol (E), relaxin (R), and progesterone (P) treatment have differential effects on the loss of collagen in fibrocartilages. Temporomandibular joint (TMJ) discs, pubic symphysis, knee meniscus, and articular cartilage were retrieved from control and hormone-treated rabbits, and the total collagen content was determined by the Sircol assay and normalized to the total dry weight for each sample. Histograms of the mean (± standard deviation) collagen concentration in sham-operated controls (SC), ovariectomized normal controls (NC), and rabbits treated with E, R, E + R (ER), P, P + R (PR), and E + P + R (EPR) groups were plotted. Ovariectomy resulted in a significant reduction of collagen content in TMJ disc **(a)** and increase collage in the pubic symphysis **(b)** but had no effect on collagen in knee articular cartilage **(c)** and meniscus **(d)**. Administration of E, R, and ER maintained the reduction of collagen content in TMJ disc produced by ovariectomy and caused a significant reduction in collagen in pubic symphysis relative to NC and in knee articular cartilage relative to SC and NC. In contrast, progesterone antagonized the effects of E, R, and ER on collagen content in TMJ disc, pubic symphysis, and the knee articular cartilage. Collagen content in knee meniscus was not affected by treatment with any of these hormones. Data were collected from a minimum of six rabbits in each group. (* < 0.05; ** < 0.01; *** < 0.001; **** < 0.0001; a, versus SC; b, versus NC; c, versus P; d, versus PR; e, versus EPR; NS, not significant.)

The TMJ disc in ovariectomized controls showed a significant loss of collagen relative to sham-operated controls (Figure 3a). This decrease in TMJ disc collagen after ovariectomies was maintained when β-estradiol, relaxin, or β-estradiol + relaxin was administered to the rabbits. In contrast, administration of progesterone alone or in combination with relaxin or β-estradiol + relaxin maintained collagen concentrations in the TMJ disc at levels found in sham-operated controls and at significantly greater levels than in ovariectomized controls. This implies that the decrease in progesterone after ovariectomy leads to collagen loss from the TMJ disc and that administration of exogenous progesterone protects these tissues from the loss of collagen.

Changes in collagen concentrations in the pubic symphysis after ovariectomies with or without administration of various hormones were very similar to those reported previously [12], validating the use of this tissue as an appropriate positive control for our studies. Ovariectomized controls had significantly higher concentrations of collagen than did sham-operated controls (Figure 3b). Administration of β-estradiol caused a significant decrease in the pubic symphyseal collagen relative to ovariectomized controls but not relative to sham-operated controls. Additionally, relaxin or β-estradiol + relaxin produced a significant reduction in collagen relative to both sham-operated and ovariectomized control rabbits. When relaxin was administered to β-estradiol-primed animals, the loss of collagen was accentuated relative to those exposed to β-estradiol or relaxin alone as evidenced by the level of significance between these groups and control groups. Administration of progesterone alone, progesterone + relaxin, or β-estradiol + progesterone + relaxin restored the collagen concentrations to, or slightly above, those found in sham-operated rabbits. Rabbits receiving progesterone alone had significantly lower concentrations of pubic symphyseal collagen than did ovariectomized controls or those administered β-estradiol + progesterone + relaxin. Finally, pubic symphyseal collagen concentrations in rabbits receiving either progesterone + relaxin or β-estradiol + progesterone + relaxin were similar to those of ovariectomized controls.

Treatments of rabbits with any one of the three hormones or any combination of these hormones produced some loss of articular cartilage collagen relative to that in ovariectomized controls (Figure 3c). Additionally, administration of β-estradiol or β-estradiol + relaxin resulted in significantly lower collagen concentrations relative to sham-operated controls or rabbits receiving progesterone alone or progesterone + relaxin.

Finally, as with GAGs, the knee meniscus did not show any changes in collagen concentrations either after ovariectomy or with the administration of any of the hormones alone or in various combinations (Figure 3d).

## Discussion

Here, we show for the first time, the contribution of relaxin and also of β-estradiol to the degradation of TMJ fibrocartilage and (to a lesser extent) of knee articular cartilage *in vivo*, the lack of potentiation of these responses to relaxin by β-estradiol, and progesterone's prevention of matrix loss mediated by β-estradiol and/or relaxin. These findings suggest that, by modulating the remodeling of the ECM of cartilage, these hormones may play an important regulatory role in the normal and pathologic metabolism of cartilaginous tissues. In the latter scenario, it is conceivable that individuals with abnormal absolute or relative levels of one or more of these hormones or their receptors might incur progressive loss of matrix macromolecules, leading to joint disorders characterized by the degeneration of specific cartilages or fibrocartilages [27]. Such potential hormone-mediated changes in the composition of the ECM can significantly impact the ability of joints to sustain and distribute mechanical loading and can also substantially affect the normal function and survival of cells in tissues [28-30].

There is a striking female preponderance for many types of joint diseases in general and for TMJ diseases in particular. TMJ disorder is an umbrella term describing a group of clinical signs and symptoms involving the masticatory musculature, the TMJ, and associated structures such as the fibrocartilaginous disc [31]. TMJ disorders are distinguished from similar diseases of other joints by one specific epidemiologic difference, namely that, unlike many similar diseases of other joints that afflict postmenopausal women, TMJ diseases are observed primarily in women of reproductive age. On the basis of our findings, it is plausible that specific reproductive hormones target this highly fibrocartilaginous joint for degradative activity by upregulating particular MMPs that contribute to the loss of collagen and GAGs [5,21]. In keeping with this postulate, our studies show that the responses of TMJ disc to relaxin, β-estradiol, and progesterone are more similar to those observed in the pubic symphysis than to those seen in the knee meniscus or articular cartilage, suggesting that the observed effects of these hormones are specific to cell type and tissue type. Additionally, the link between the relaxin- and β-estradiol-mediated induction of MMPs and the loss of collagen and GAGs in the TMJ disc by these hormones has been demonstrated previously in our tissue explant studies. However, the *in vivo *induction of MMPs by these hormones and the association between MMP induction and matrix loss remain to be established.

The reasons for the observed differences in responsiveness of the TMJ disc fibrocartilage, knee meniscus fibrocartilage, articular cartilage, and pubic symphysis to the hormones are not known. However, our recent work on identifying and quantifying estrogen receptor-α and -β and relaxin receptors LGR7 (leucine-rich repeat-containing, G protein-coupled receptor 7) and LGR8 may provide some insights into one potential reason for the observed differences. These findings show varied expression of these receptors between these tissues, supporting the conclusion that the more robust responses of the pubic symphysis and TMJ disc to relaxin and β-estradiol are possibly related to the presence of higher levels of receptors in these tissues than in the knee meniscus [32]. The findings of the current study also raise the possibility that the distinct composition, organization, and biomechanical characteristics in different subtypes of cartilages [22] may influence the ability of systemic hormones to access each of the tissues, thereby influencing the net amount of matrix loss. These concepts need further study.

The similarities of our findings on the hormone-mediated changes in the collagen content of the pubic symphysis with those of previous investigators [12,13] suggest that our model has several commonalities to animal models used previously and attest to its relevance for the purposes of this study. Samuel and colleagues [12], for example, showed that relaxin causes 64% (± 4%) and 68% (± 6%) decreases, respectively, in pubic symphysis collagen in unprimed and β-estradiol-primed ovariectomized non-pregnant rats. This compares with relaxin contributing to collagen loss of approximately 60% in unprimed and 80% in β-estradiol-primed rabbit pubic symphysis in the present study. Also, in agreement with our observations on the pubic symphysis, previous findings show that progesterone treatment rescues the collagen loss mediated by relaxin in estrogen-primed animals [12,16]. Together, these observations indicate that the pubic symphysis served as an appropriate positive control for our experiments. In addition to confirming these previous observations on relaxin's contribution to collagen loss in the pubic symphysis, we also show new findings that β-estradiol and relaxin cause a slight, but statistically significant, loss of GAGs from this tissue.

Whereas a few previous studies have demonstrated other non-reproductive target sites, including the *in vivo *matrix turnover of lung alveolar tissues [33] and the *in vitro *modulation of tissue degradation in dermal fibroblasts [34] as well as fibrocartilaginous cells or tissues from joints [5,21], our findings show for the first time that relaxin alters matrix composition of specific cartilages from non-reproductive sites *in vivo*. As with findings on reproductive tissues [9,12,16,17,22], we had previously demonstrated that the effect of relaxin on MMP induction is potentiated by β-estradiol in isolated fibrocartilaginous cells from the TMJ [5]. In contrast, TMJ fibrocartilaginous explants [21] and the current *in vivo *findings show no potentiation by β-estradiol of relaxin's induction of MMPs or loss of collagen and GAGs. These differences in responses between cultured cells and tissue explants can likely be attributed to the differences in the behavior of isolated cells from cells in their natural matrix environment within the context of the explants and in the intact animal.

Our results also show a significant increase in systemic relaxin levels in all groups administered relaxin, with median concentrations ranging from a low of 37 pg/ml for rabbits receiving progesterone + relaxin to a high of 58 pg/ml for those administered β-estradiol + relaxin. This range of concentrations of relaxin in all groups of animals administered this hormone alone or in combination with other hormones is similar to that found systemically in cycling women [35-37]. Interestingly, our findings showed that β-estradiol priming enhances systemic relaxin concentrations, whereas priming with progesterone tended to diminish the serum levels of relaxin. These effects of hormone priming on systemic relaxin levels were reflected in the statistically greater fold increase of relaxin in β-estradiol + relaxin versus progesterone + relaxin groups (*p *< 0.01) (Figure 1a). This finding corresponds to some degree with the decreases in collagen and GAGs mediated by relaxin and β-estradiol + relaxin, but not by progesterone, progesterone + relaxin, or β-estradiol + progesterone + relaxin in the more responsive of the tissues studied, namely the TMJ disc and pubic symphysis. These results suggest that administration of relaxin using an implanted subcutaneous osmotic pump contributes to the increases in systemic relaxin concentration in which β-estradiol and progesterone may act as regulators of systemic relaxin concentration. However, further studies are required to clarify the mechanism by which β-estradiol, relaxin, and progesterone interact with each other to modulate systemic relaxin levels and subsequently maintain or disturb the homeostasis of the ECM in fibrocartilage.

## Conclusion

Our study demonstrates a cross-talk between relaxin, β-estradiol, and progesterone that may affect systemic relaxin concentrations and ultimately the net matrix remodeling activity of these hormones alone or in various combinations in target tissues *in vivo*. We also show specificity in the responses of various cartilages and fibrocartilages to modulation of matrix loss by relaxin and β-estradiol. Excessive degradation of collagen and GAGs by relaxin and/or β-estradiol may perturb the homeostasis of ECM in these joints and eventually contribute to the degenerative disease of the target joint.

## Abbreviations

DMMB = dimethylmethylene blue; ECM = extracellular matrix; GAG = glycosaminoglycan; LGR = leucine-rich repeat-containing, G protein-coupled receptor; MMP = matrix metalloproteinase; OD = optical density; TMJ = temporomandibular joint.

## Competing interests

The authors declare that they have no competing interests.

## Authors' contributions

GH performed animal procedures, including hormone injections and implantation of osmotic pumps, performed collagen and GAG assays, and compiled the data. QZ assisted GH in the above animal procedures and performed the relaxin enzyme-linked immunosorbent assays. JC performed all initial studies to establish the model and also helped to establish the experimental procedures for sample collection and assays. TH performed the surgeries to retrieve all the tissues and blood from the animals. WW assisted SK in data analysis and contributed to the interpretation of the data and preparation of the manuscript. SK conceived, designed, and supervised the study, performed the statistical analysis, and contributed to the interpretation of the data and the revision of the manuscript. All authors have read and approved the final manuscript.
